# Trait-like nocturnal sleep behavior identified by combining wearable, phone-use, and self-report data

**DOI:** 10.1038/s41746-021-00466-9

**Published:** 2021-06-02

**Authors:** Stijn A. A. Massar, Xin Yu Chua, Chun Siong Soon, Alyssa S. C. Ng, Ju Lynn Ong, Nicholas I. Y. N. Chee, Tih Shih Lee, Arko Ghosh, Michael W. L. Chee

**Affiliations:** 1 Sleep and Cognition Laboratory, Centre for Sleep and Cognition, Yong Loo Lin School of Medicine, National University of Singapore, Singapore, Singapore; 2 Laboratory of Neurobehavioral Genomics, Neuroscience and Behavioral Disorders Programme, Duke-NUS Medical School, Singapore, Singapore; 3 Institute of Psychology, Leiden University, Leiden, The Netherlands

**Keywords:** Risk factors, Signs and symptoms

## Abstract

Using polysomnography over multiple weeks to characterize an individual’s habitual sleep behavior while accurate, is difficult to upscale. As an alternative, we integrated sleep measurements from a consumer sleep-tracker, smartphone-based ecological momentary assessment, and user-phone interactions in 198 participants for 2 months. User retention averaged >80% for all three modalities. Agreement in bed and wake time estimates across modalities was high (rho = 0.81–0.92) and were adrift of one another for an average of 4 min, providing redundant sleep measurement. On the ~23% of nights where discrepancies between modalities exceeded 1 h, k-means clustering revealed three patterns, each consistently expressed within a given individual. The three corresponding groups that emerged differed systematically in age, sleep timing, time in bed, and peri-sleep phone usage. Hence, contrary to being problematic, discrepant data across measurement modalities facilitated the identification of stable interindividual differences in sleep behavior, underscoring its utility to characterizing population sleep and peri-sleep behavior.

## Introduction

Sleep is increasingly recognized as a major modifiable lifestyle risk factor and this has contributed to a boom in sales of consumer wearables that track it. In the short term, poor sleep is associated with impaired cognitive performance, mood, and motivation^1–4^, while over extended periods, sleep loss increases the risk of diabetes mellitus, hypertension, cardiovascular and cerebrovascular disease, earlier cognitive decline, Alzheimer’s Disease, and depression^5,6^. Many epidemiological associations have been derived using self-report questionnaires which are subject to recall errors and are inconvenient for the collection of longitudinal data. Objective measurement of sleep is thus desirable for longitudinal or long-term assessment of sleep duration, timing, regularity, and continuity.

Polysomnography (PSG) while accurate is expensive, labor-intensive, and often limited to short study durations. Research-grade actigraphy is less expensive, relatively unobtrusive, and can be conveniently deployed for longer durations. However, most actigraphs require laboratory sited download from the devices. The recent proliferation of commercially available consumer wearable and smartphone technologies opened up an opportunity to collect remote sleep and health data, with a highly reduced need for participant contact and intervention. Accordingly, an increasing number of studies have turned to such methods for long-term ambulatory tracking of sleep^7–11^.

Wearable sleep trackers measure body motion, heart rate, and skin temperature or some combination of these and provide periodic feedback about user behavior through appealing graphs and charts. These inexpensive devices hold promise for collecting large-scale longitudinal sleep data that could dramatically improve the characterization of longitudinal sleep patterns compared to cross-sectional estimates of sleep duration or quality obtained from questionnaire data. While less accurate than PSG, collecting data from such devices is far more economical, technically simple, ecologically valid, and can be obtained for multiple nights of sleep in the participants’ home^12–14^. Data collected from such devices have found associations between poorer sleep and surrogates of cardiovascular and metabolic health as well as telomere attrition^15,16^.

A key component of the wearable sleep tracker ecosystem is the smartphone, which serves both as a portal for gathering and storing information, and a means for providing feedback on recorded behavior, tips on improvement, and motivation to act on these recommendations^17^. Along with the expansion of indirect measurement of sleep through wearable trackers, there are novel means to characterize peri-sleep behaviors by observing the timing and frequency of smartphone usage and the temporal features of user-touchscreen interaction (“tappigraphy”)^7,18–21^. This methodology unobtrusively informs about activities that could interfere with sleep while assisting in sleep measurement^18,22^. Tappigraphy has also uncovered associations between sleep patterns and mental health^21,23,24^. Moreover, smartphone interactions while the user is otherwise lying still in bed may detect periods of wake where motion is below the threshold set for identification of wakefulness by wearable devices.

A further advantage of smartphones is that they can serve as a convenient modality to collect self-reported measures from participants. Tracking of sleep quality, as well as associated mood through ecological momentary assessment (EMA), can provide vital information about mental wellbeing, and serve as modern-day sleep diaries that cannot so easily be misplaced or forgotten as compared to conventional diary^25–27^.

A common approach for garnering acceptance of the use of wearable and smartphone-based technologies for research is to compare their sleep measurement performance to PSG and research actigraphy. Such comparisons are important for benchmarking the accuracy of each device, but they do not highlight the merits of using less accurate but more convenient and economical methods to assay sleep regularity, timing, or the factors influencing these objectively on a large scale. As such, a different approach for assessing sleep behavior is to collect data on the same individual using multiple sensing techniques. The fusion of information thus obtained can provide redundant information whose concordance as well as discordance might inform about sleep behavior. In the current study, we tested the utility of this approach in two related ways. First, we assessed the level of agreement between sleep estimated from different modalities. Second, we examined patterns of discrepancies where different modalities provide diverging information. Concordant information is useful when one source of information is missing (e.g., when the participant did not wear the device, or the device ran out of battery). Discordant information as demonstrated here proved to be informative as well, as it carries information on specific sleep-related behaviors. We combined data from three sleep tracking systems: (1) wearable sleep tracker (Oura ring), (2) background logging of human–smartphone interactions (Tappigraphy), and (3) self-report via phone-based daily questionnaires (EMA) to characterize sleep and perisleep behavior^28^. A sample of young to middle-aged adults was tracked over a period of 2 months.

## Results

### High rates of user acceptability and retention

**Fig. 1:**
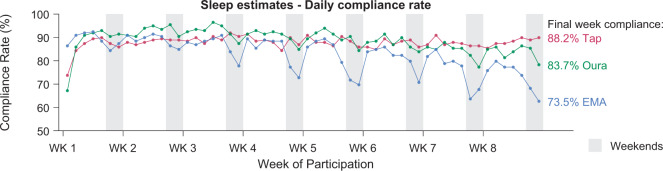
Data completion over time. Daily compliance rate for tappigraphy (Tap; pink curve), Oura (green curve), and self-reported EMA (blue curve). Weekends are delineated as grey-shaded regions. Average daily compliance rates for each modality during the final week of subjects’ participation are also indicated.

Wearable, phone-based, and self-report sleep data were obtained from 198 university students and staff (age = 26.20 ± 5.83 years, 61 males, 78 staff), over a period of 8 weeks. Of 11,088 potential nights (summed across all 198 subjects over the 8-week protocol), sleep was recorded from the Oura wearable device on 9825 nights (89%), via smartphone interaction tracking (Tappigraphy) on 9740 nights (88%), and from self-reported EMA on 9166 nights (83%; refer to Table 1 for information about sleep variables obtained from each modality). On 7581 nights (68.4%) concurrent data from all three modalities was available. Retention over time was consistently high for wearable and smartphone-based tracking (Fig. 1), while EMA-based daily self-reports showed a gradual decline over time from the third week of subjects’ participation (EMA: *F*(7,1379) = 27.72, *p* < .001, *η*_p_^2^ = 0.123). Also, higher completion rates were observed on weekdays vs. weekends for Oura and EMA (Oura: *F*(1,197) = 7.47, *p* = 0.007, *η*_p_^2^ = 0.037; EMA: *F*(1,197) = 60.22, *p* < 0.001, *η*_p_^2^ = 0.234). Completion rates were consistently high for tappigraphy (no decline over time, *F*(7,1379) = 1.077, *p* = 0.38, *η*_p_^2^ = 0.005). Overall compliance rates remained above 70% even during the last week of monitoring.

**Table 1:** Sleep estimates obtained from each modality.

Summary variable	Oura	Tappigraphy	EMA
Nights completed	9825	9740	9166
Completion rate (%)	88.6	87.8	82.7
	Mean (SD)	Mean (SD)	Mean (SD)
Bedtime (hh:mm)	01:40 (01:53)	01:23 (01:57)	01:31 (01:53)
Wake time (hh:mm)	09:22 (02:01)	09:06 (01:54)	09:05 (01:48)
Midsleep time (hh:mm)	05:31 (01:48)	05:14 (01:43)	05:18 (01:42)
Time in bed (TIB) (hh:mm)	07:43 (01:27)	07:43 (01:43)	07:34 (01:27)
Wake after sleep onset (min)	44.8 (32.8)	–	–

### Agreement in sleep estimation across modalities

**Fig. 2:**
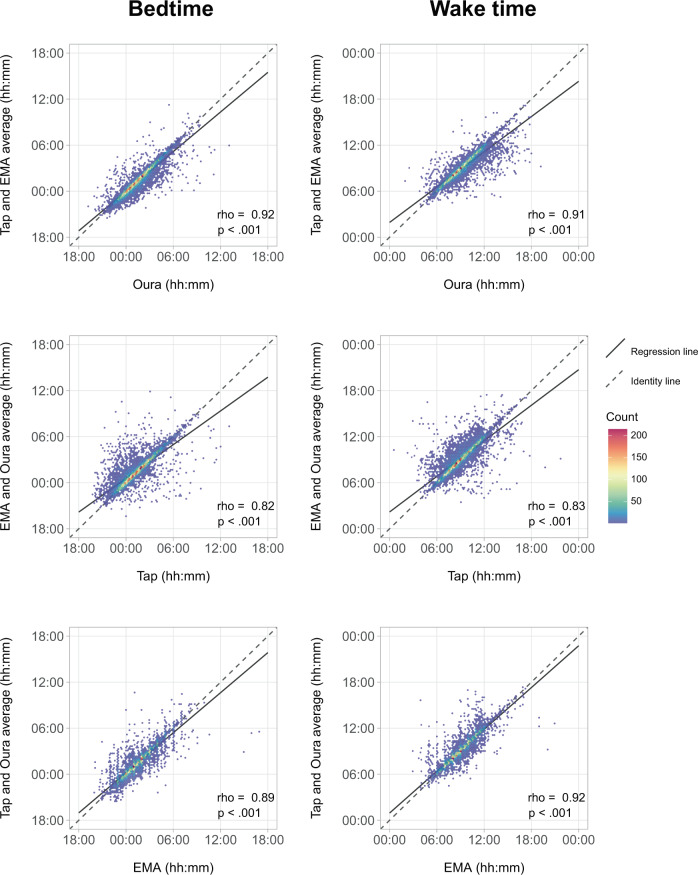
Agreement of sleep estimates between modalities. Density scatters plot of the bed (left panel) and wake time (right panel) estimates from each modality (*x*-axis) against estimates obtained from the average of the other two modalities (*y*-axis). Correlation coefficients are stated for each of the subplots (nights with all three modalities, *n* = 7581). The dashed line indicates the identity line where there is complete agreement between modalities.

To determine how well sleep measures agreed across the different modalities, we ran pairwise correlation analyses for each modality’s sleep estimates and the averaged estimates of the remaining two. There was good agreement across the three methods of sleep assessment (Fig. 2) with correlations highest for wearable and EMA modalities (Spearman’s rho = 0.89–0.92, *p*’s < 0.001), and slightly lower for Tappigraphy (Spearman’s rho = 0.82–0.83, *p* < 0.001). Scatter plots in Fig. 2 show that on most of the nights, observed bed and wake time estimates were concentrated around the line of identity (i.e., showing highly similar estimates between modalities).

### Inter-modality discrepancies

To examine whether there were systematic discrepancies between sleep estimation methods, sleep estimates from the Oura ring and Tappigraphy tracking app were referenced against EMA self-reported sleep times. This approach was taken as most epidemiological data on the association between sleep duration and health has been derived from self-reported sleep durations. Oura bedtime estimates were later by 3.5 min (*z* = 27.17, *p* < 0.001) and wake time estimates were later by 4.3 min (*z* = 44.43, *p* < 0.001) on the median. Tappigraphy showed a slightly earlier bedtime of 1.9 min (*z* = −12.41, *p* < 0.001) and a later wake time of 1.4 min (*z* = 8.57, *p* < 0.001) relative to self-reports. In sum, high agreement among modalities can be seen on a majority of the nights with average discrepancies being around 5 min (see Table 2).

**Table 2:** Comparison of sleep estimates with self-reported EMA as reference.

Modality	Statistics	Bedtime (hh:mm)	Wake-time (hh:mm)
Oura (*n* = 8459)	Mean	01:37	09:19
	Median	01:28	09:04
	IQR	02:16	02:26
EMA (on corresponding nights)	Mean	01:28	09:04
	Median	01:15	09:00
	IQR	02:15	02:05
Tap (*n* = 8146)	Mean	01:19	09:04
	Median	01:10	08:54
	IQR	02:20	02:25
EMA (on corresponding nights)	Mean	01:28	09:04
	Median	01:15	09:00
	IQR	02:15	02:05

### Highly discrepant patterns

**Fig. 3:**
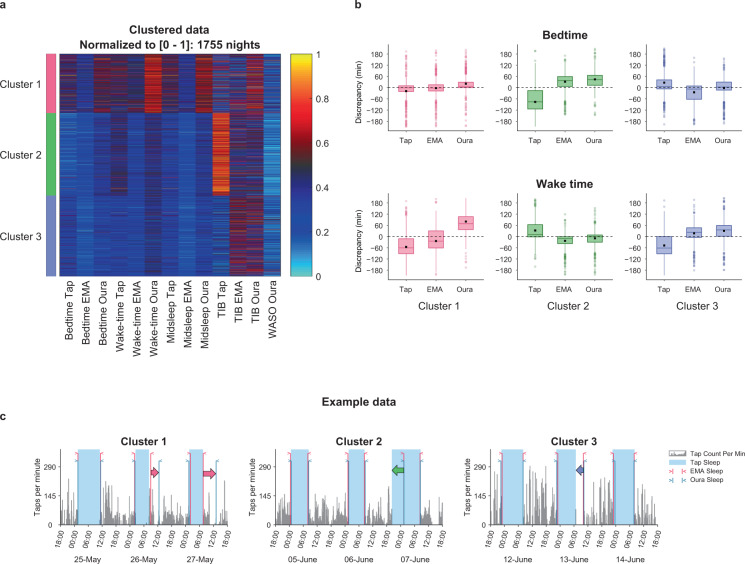
Identifying patterns of modality discrepancy through clustering. **a** Clustering of discrepant nights based on bedtime, wake time, midsleep time and time in bed (TIB) obtained from each modality along with WASO from Oura (*n* = 1755, discrepancy >1 h). Warm colors indicate later sleep timings (bedtime, wake-time, and midsleep) and longer durations (TIB, WASO) normalized per sleep metric, and cool colors indicate earlier sleep timings and shorter durations. **b** Plots show the discrepancy patterns for the resulting clusters for bed and wake time metrics. Estimates from each modality were compared to the overall average of the three modalities. Each boxplot displays the mean (black square) and the median (centerline) discrepancy values. The upper and lower hinges correspond to the first and third quartiles, and the upper and lower whiskers extend to the furthest point within 1.5 times the interquartile range from the hinges. **c** Illustrative samples of daily phone tap activity with sleep timings from the three modalities for each high-discrepancy cluster. Cluster 1: delayed Oura wake times (26th/27th May). Cluster 2: earlier tappigraphy-based bedtime relative to Oura and EMA (7th June). Cluster 3: earlier tappigraphy-based wake time (13th June).

While average inter-modality discrepancies were small, inter-modality discrepancies of >1 h were observed on 1755 nights (23% of recorded nights). To gain insight into the sources of these discrepancies, we performed *k*-means clustering on the basis of 13 sleep features taken from the different modalities (Fig. 3). We included four features for each of the three modalities (bedtime, wake time, midsleep time, and time in bed (TIB)), as well as one feature that was only available from Oura (wake after sleep onset [WASO]). Three distinct clusters were identified, each with a specific profile of discrepancy across modalities (Fig. 3b). These cluster solutions remained stable when a range of different discrepancy thresholds was used (1.5, 2, and 2.5 h; see Supplementary Fig. 1 and Supplementary Table 1).

Cluster 1 (*n* = 467) consisted of nights with delayed sleep timings across all modalities and later Oura wake times relative to the other clusters. From the corresponding discrepancy data, we observed that Oura’s wake time estimates for this cluster were later in comparison to the other modalities (Fig. 3b). On a Cluster 1 night, EMA wake time coincided with Tap-based wake time (Fig. 3c), while the Oura identified wake time was delayed. A possible explanation for this is that subjects were using their phones in bed after awakening, but lying relatively still.

Nights in Cluster 2 (*n* = 651) generally had relatively longer tappigraphy estimated TIB associated with earlier bedtimes and later wake times. On such nights, there was good agreement in bed and wake times determined by EMA and Oura, while tappigraphy-based sleep duration was longer (Fig. 3b, c). This may possibly reflect early cessation of phone use before going to sleep and later commencement of phone use post-awakening than with participants of the other two clusters.

On Cluster 3 nights (*n* = 637), Oura and EMA determined TIB was longer in comparison to Tappigraphy. On the other hand, Tappigraphy assessed relatively later bedtimes and earlier wake times compared to the other two modalities. Tappigraphy-determined wake time was earlier relative to EMA and Oura (Fig. 3b). In the example, a short period of tap activity was flanked by periods of inactivity (Fig. 3c). This likely corresponds to brief awakening(s) accompanied by short phone use (potentially to snooze an alarm or to check for messages), after which sleep was resumed.

### Individual phenotyping based on discrepancy clusters

**Fig. 4:**
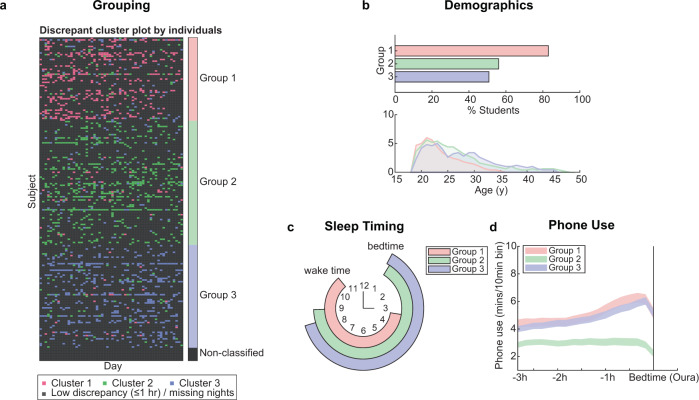
Individual phenotyping based on discrepancy clusters. **a** Distribution of high-discrepancy nights over individual participants (rows) plotted for each day (columns) of the 8-week monitoring period. High-discrepancy nights were grouped according to cluster membership. **b** Percentage students, and age distribution of resulting groups. **c** Sleep characteristics as quantified on low-discrepancy nights. **d** Pre-bedtime phone usage (Tappigraphy derived) in the window 3 h prior to bedtime (Oura defined). Shaded areas depict mean ± 1 SEM.

When examining the distribution of discrepancy patterns across individuals, a high within-individual consistency was observed (see Fig. 4). Most participants expressed a dominant type of discrepancy over the other two (median percentage of dominant cluster per subject = 84.41%, IQR = 33.33%; see Supplementary Fig. 2 and Supplementary Table 2). Fourteen participants had an equal contribution of two or three discrepancy patterns, and eight participants did not have any nights with discrepancies greater than 1 h. These individuals were excluded from the following analysis. The remaining individuals were classified according to their dominant discrepancy cluster pattern and resulting groups were compared (Table 3), in order to identify demographic and behavioral factors associated with these discrepancy patterns.

**Table 3:** Comparing groups on sleep, sociodemographic, smartphone usage, and daily well-being.

Variable of interest	Group 1 *N* = 47^a^	Group 2 *N* = 66^a^	Group 3 *N* = 63^a^	Test statistics	*p*
	Mean (SD)	Mean (SD)	Mean (SD)		
Number of discrepant nights	10.7 (8.3)	9.6 (8.3)	8.8 (8.3)	*F*(2,173) = 0.66	0.52
Sleep parameters^b^					
Bedtime (hh:mm)	03:18 (01:08)^c,d^	01:04 (01:11)^e^	00:57 (00:58)^e^	*F*(2,173) = 75.20	<0.001
Wake time (hh:mm)	10:41 (01:08)^c,d^	08:58 (01:09)^e,d^	08:28 (00:59)^e,c^	*F*(2,173) = 58.85	<0.001
TIB (hh:mm)	07:23 (00:52)^c^	07:55 (00:46)^e,d^	07:31 (00:45)^c^	*F*(2,173) = 7.37	<0.001
WASO (min)	44.0 (23.2)	38.8 (18.2)	45.1 (25.0)	*H*(2) = 1.47	0.48
Sleep efficiency (%)	85.9 (5.1)	87.6 (3.9)	85.8 (5.7)	*H*(2) = 3.39	0.18
Sociodemographics					
Age in years	23.8 (3.7)^c,d^	26.6 (6.3)^e^	27.7 (6.3)^e^	*F*(2,173) = 6.48	0.002
Sex—% Females	63.8	72.7	66.7	*χ*^2^(2) = 1.11	0.58
Occupation (% staff)	17.0^f^	43.9	49.2^g^	*χ*^2^(2) = 13.01	0.001
Smartphone usage					
Median daily tap count	12,735 (7056)^c^	7130 (4625)^e,d^	10,461 (5324)^c^	*H*(2) = 26.33	<0.001
Median daily device use (min)	418.3 (165.9)^c^	255.4 (121.5)^e,d^	392.2 (145.0)^c^	*H*(2) = 35.79	<0.001
Daily device usage 1 h before Oura bedtime (min)	35.5 (13.9)^c^	17.1 (11.7)^e,d^	33.5 (10.9)^c^	*F*(2,173) = 42.34	<0.001
Sleep without phone (%)^h^	17.2	45.5^g^	22.2	*χ*^2^(2) = 8.60	0.014
Daily self-reported well-being					
Sleep quality (1–5)	3.6 (0.5)	3.6 (0.5)	3.6 (0.6)	*F*(2,173) = 0.15	0.86
Morning sleepiness (0–100)	51.4 (15.2)^c,d^	40.3 (18.0)^e^	42.6 (16.2)^e^	*F*(2,173) = 6.51	0.002
Morning mood (0–100)	50.4 (9.7)^d^	55.7 (15.6)	57.3 (12.5)^e^	*H*(2) = 11.96	0.003
Evening mood (0–100)	54.9 (9.1)	56.4 (14.8)	57.3 (11.4)	*F*(2,173) = 0.51	0.60
Morning stress (0–100)	41.1 (19.7)	41.9 (20.4)	44.7 (19.0)	*F*(2,173) = 0.54	0.58
Evening stress (0–100)	42.2 (18.9)	40.0 (19.3)	43.4 (18.5)	*F*(2,173) = 0.54	0.59

^a^*N* = 22 participants had no dominant cluster pattern, or had no nights with discrepancies >1 h, and were excluded from group analysis.

^b^Values based on nights with <1 h discrepancy averaged across modalities.

^c^Measure significantly different from Group 2.

^d^Measure significantly different from Group 3.

^e^Measure significantly different from Group 1.

^f^Observed proportion for cell significantly lower than its expected proportion.

^g^Observed proportion for cell significantly higher than its expected proportion.

^h^This item was completed by *N* = 118 participants.

For comparing sleep variables among groups, we excluded the *n* = 1755 high-discrepancy nights that were used for the original high-discrepancy profiling. This left a total of *n* = 5826 (low-discrepancy) nights. Sleep estimates for those nights were averaged across all three modalities. This analysis showed that individuals from Group 1 (*N* = 47), mostly slept in accordance to Cluster 1 nights (76.6% of high-discrepancy nights, characterized by delayed in Oura-determined wake time), had later bedtimes (compared to Group 2: 134 min, Group 3: 141 min, both *p*’s < 0.001) and wake times (Group 2: 102 min, Group 3: 133 min, both *p*’s < 0.001) on nights with less than 1 h discrepancy in cross-modality sleep timing estimates. In comparison to Group 2, Group 1 had shorter TIB on these nights (Group 2: −32 min, *p* = 0.001). Individuals in Group 1 tended to be younger compared to the other two groups and were predominantly students (see Table 3).

Members of Group 2 (*N* = 66) had mostly Cluster 2 nights (82.7% of high-discrepancy nights; characterized by longer tappigraphy assessed TIB). This group had less daily smartphone use (Group 1: −163 min, Group 3: −137 min, both *p*’s < 0.001) and lower daily tap count (Group 1: −5605, Group 3: −3331, both *p*’s < 0.001). Furthermore, a greater proportion of Group 2 individuals reported that they did not usually sleep with their phones near them (see Table 3). This group had the longest TIB, even for low-discrepancy nights (7.91 h).

Group 3 individuals (*N* = 63) had most of their discrepant nights belonging to Cluster 3 (86.4%), where morning sleep seemed to be interrupted with a brief period of phone use before getting back to sleep. This group was over-represented by staff (49.2%). They had bedtimes resembling that of Group 2 (*p* = 0.83) but earlier wake times (Group 2: −31 min, *p* = 0.02), and shorter TIB (Group 2: −24 min, *p* = 0.01). In terms of smartphone habits, Group 3 matched Group 1 (tap count: *p* = 0.31, device use: *p* = 0.79), and had longer daily device use and higher daily tap count as compared to Group 2. Group 3 individuals reported better mood in the morning as compared to those in Group 1.

## Discussion

We combined sleep measurement using wearable sleep tracking with smartphone based tappigraphy and EMA to provide redundant as well as complementary information about sleep behavior. There was a high level of data provision (>80% average) over 8 weeks which bodes well for large-scale longitudinal studies. The high compliance rates could be due to participants being incentivized incrementally for regular data logging (see Methods), combined with a relatively low burden of data collection through wearable and phone tracking. Overall, agreement between the three modalities was good, supporting the utility of gathering redundant data in long-term studies where participants will occasionally fail to provide information from one modality. On the minority of nights where significant discrepancy across modalities did occur, the patterns of these, cross-referenced with demographic, questionnaire, and phone use intensity data, provided interesting insights into stable differences in sleep behavior across individuals.

The high completion rates underline the feasibility of a multi-sensor approach for long-term sleep tracking, particularly when participant effort required to provide data is lower as in the case with wearable and tappigraphy based monitoring. Importantly, sleep estimates from the three modalities showed high correlation (ranging from rho = 0.82 to rho = 0.92, with median discrepancies in sleep duration estimation around 4 min). Given these findings, there seems to be considerable promise in the current approach of combined deployment of wearable, and mobile phone-based sleep tracking. The possibility for regular cloud-based data transfer and remote monitoring facilitates data collection with a minimal need for lab visits or interruption of daily routines. This may allow for the extension of sleep tracking for several months or even years. Furthermore, the relatively lower cost of consumer-grade wearable devices compared to research-grade actigraphy (or PSG) enhances the scalability of this approach. Recent validation studies have reported that the performance of the Oura ring for measurement of sleep timing and duration was comparable to that of research actigraphy^29–31^ (see “Methods”), while Tappigraphy shows a high correlation with actigraphy^18^.

Consumer wearables carry some disadvantages. At the present time, researchers do not have recourse to re-analyze raw data and scoring algorithms are trade secrets. Failure to synchronize the updating of sleep measurement algorithms can impair the collection of long-term longitudinal data although this issue is being addressed by some manufacturers. Data confidentiality differs across manufacturers.

The most novel aspect of the current multi-modal approach lies in the insights one can derive from examining discrepancies between measurements from each of the three modalities. Normally, such discrepancies relate to undesirable, modality-specific deficits in sleep or wake detection. However, in our data this turned out to be informative in that clustering of high-discrepancy nights revealed three distinct patterns of sleep and peri-sleep behavior. Moreover, each of these patterns consistently mapped onto individual participants, resulting in three corresponding groups.

The first group comprised younger individuals (mostly students) with late bedtimes, short time in bed, and active phone use in the morning after waking. Morning phone use, as detected through tappigraphy, preceded wearable-detected wake time on high discrepant nights. It therefore may potentially reflect in-bed phone usage with low levels of body movement (below a threshold level of physical activity)^18^. The very late bedtimes observed here are likely related to data being collected during the lockdown period of the 2020 COVID-19 pandemic. Robust shifts to later sleep timing have been reported during such lockdowns^14,32,33^. In addition, the pattern of later sleep and morning phone use (presumably in-bed), could be related to online study activities (e.g., attending online lectures) as learning was completely shifted online, and commuting to campus was not allowed^34^. A previous study that concurrently measured sleep and phone use^7^, has suggested that a pattern of e-device use in bed might be related to higher sleep inertia due to sleep restriction^35,36^. Interestingly, this group reported the worst morning mood, and highest sleepiness, making this a potential target group for sleep improvement intervention.

A second group was characterized by lighter phone use than other individuals, logging about 4.5 h of active phone use a day (versus 6.5 to 7 h for the other groups). In addition, these individuals showed lower phone use before bedtime (17 min in the hour before bedtime), and a lower proportion of these individuals reported bringing their phones to bed, compared to the other two groups. This pattern is interesting because restricting peri-sleep electronic device screen time is often seen as a means to improve sleep^37,38^. Heavy phone use before bedtime is associated with poorer sleep quality and quantity and with increased risk of mental health issues (e.g., depression)^37,39–41^. Although we tracked only smartphone usage and cannot exclude the usage of other e-devices in bed, the observation that this group had the longest TIB suggests that not using one’s phone before bed can lengthen nocturnal sleep duration^42,43^. As such restricting phone use before bedtime could form part of an effective program to improve sleep, wellbeing, and next-day performance^38,44–46^.

A third group comprising older participants had the highest proportion of working adults, with time in bed that was intermediate between that of Groups 1 and 2. These participants had sleep records showing a brief period of phone activity followed by resumption of sleep in the morning. This is suggestive of checking the time or looking at incoming messages or e-mail. While isolated episodes might be inconsequential, when occurring more frequently or repeated over multiple nights such behavior could disturb and shorten nocturnal sleep^43,47^ and may reflect the inadequate setting of rest/non-rest time management boundaries^48^.

The trait-like nature of membership in each of the discrepancy-identified groups speaks to the possibility of using such information to classify sleep and perisleep behavior beyond using the information provided by any modality alone. Fusion of sleep measures taken over extended periods when enriched with other data relevant to sleep (e.g., timing, intensity, and reason for e-device use, timing, and intensity of physical activity) opens the door to crafting individualized interventions/advice for sleep and 24 h activity patterns.

In sum, our report signals the potential of remote multi-sensor sleep tracking. The relatively high compliance rates and good levels of agreement between the different sensors indicate the utility of having redundant sleep measurement. On a minority of nights, inter-modality discrepancy patterns facilitated the characterization of different behavioral phenotypes associated with sleep and peri-sleep behaviors that could be targeted for customized sleep behavior interventions.

## Methods

### Ethics declaration

All procedures were approved by the Institutional Review Board of the National University of Singapore (NUS-IRB Ref Code: N-20-039), and all participants signed written informed consent before commencing the study.

### Participants and procedures

Two hundred university staff and students were recruited in four, weekly batches to take part in an 8-week sleep and well-being tracking study during the COVID-19 lockdown. Data reported here were collected from 27 April till 12 July 2020 (start of batch 1 till the end of batch 4), overlapping with the lockdown (7 April–1 June). Two subjects withdrew mid-study, resulting in a remaining sample of 198 (age = 26.20 ± 5.83 years, 61 males, 78 staff). Sleep was tracked using three separate modalities: (1) a sleep and activity tracking wearable device (Oura Ring Heritage; Oura Health Oy, Oulu, Finland), (2) a smartphone app tracking touchscreen interactions (Tappigraphy), and (3) self-reports through EMA (see Supplementary Table 3 for details).

Participants were incentivized to log their sleep and well-being data based on weekly completion of at least (1) 4 days of Oura tracking, (2) one day of smartphone recording (more than 1000 detected taps in a day or greater than 75% of subjects’ average daily tap count, whichever was lower), and (3) eight sessions of EMA. Subjects were given $10 reimbursements weekly based on their compliance, and a $20 study bonus upon completion of the entire study. This incentive structure was designed to encourage regular data logging across all modalities since the only completion of all three criteria resulted in weekly reimbursement. Participants were updated weekly by email on their completion rates in the preceding week. Further email assistance was offered in case of technical problems or late data syncing after completion calculation (to update completion and reimbursement rates). The use of incentivization and regular check-ins has been recommended to sustain compliance in intensive longitudinal testing studies^49^. Depending on study duration, testing intensity, and population, different incentive schemes may be effective (e.g., fixed-rate incentive and lottery-based incentive^50,51^).

Subjects were also requested to complete periodic questionnaires every 4 weeks that asked about their smartphone usage habits, stress levels, and routine. Only selected data from questions regarding smartphone usage (i.e., sleeping with the phone next to them) are included here.

### Sleep and activity tracking ring (Oura)

The Oura ring tracks heart rate, temperature changes, and movement through photoplethysmography sensors, temperature sensors, and an accelerometer to infer sleep and daytime activity. Participants were instructed to wear the ring at all times (both during day and night) and sync the data to the Oura phone app daily. Furthermore, they were instructed to charge the ring every 4–5 days. Sleep and wake periods were classified by the Oura Health algorithm based on activity and physiological data. A minimum of 3 h was required for Oura Health’s algorithm to consider a rest period as a possible sleep episode. Daily estimates for bedtime, wake time, time-in-bed (TIB), WASO, and sleep efficiency were extracted from Oura Health’s cloud API. To ensure consistency across modalities, sleep episodes exceeding 13 h were removed from the analysis.

Several recent validation studies have evaluated the performance of the Oura ring in comparison to PSG and/or actigraphy. Overall, the accuracy of sleep–wake detection was good. Two studies found no systematic error in TST estimates, with only a small absolute error as compared to PSG (87.8% of nights within 30 min error)^30^ and ambulatory EEG (7.39% mean absolute percentage error^31^). Two other studies reported modest but significant overestimation of Oura-derived TST by about 15 min, compared to PSG^52^, and actigraphy^29^ in adults, while another study reported substantial underestimation of TST compared to PSG, in an adolescent population (32–47 min)^53^. Importantly, epoch-by-epoch analysis has demonstrated high sleep-wake detection accuracy (accuracy = 0.89; sensitivity = 0.89–0.93; specificity = 0.41–0.89)^30,52,53^, comparable to actigraphy in most cases^52,53^. While Oura additionally provides sleep staging estimates (REM, deep sleep, and light sleep), these estimates are generally found to be less accurate (0.51–0.83)^30,53^. We, therefore, did not include sleep staging metrics into our analyses.

### Smartphone touchscreen interactions

Mobile phone use was recorded via a smartphone app, TapCounter^54^, to track touchscreen interactions (“tappigraphy”) and screen on/off events. Each touchscreen interaction was recorded as a timestamped event along with the active app. TapCounter operates in the background and requires minimal user intervention to function once relevant permissions were granted. From the resulting data, estimates of total daily phone use, screen-interaction count (tap count), and sleep timing were extracted following an algorithm outlined in Borger^18^. Touchscreen interaction time series were converted to 1-min epochs of binary active and inactive states based on the presence or absence of detected taps. Subsequently, a cosinor analysis^55^ was performed to capture users’ daily smartphone usage rhythm for the identification of potential rest periods; 6 h of lowest estimated tap activity in a 24 h window. Next, potential sleep episodes were identified based on gaps in actual tap activity; defined as more than two hours of near absent tap activity (less than 2 min of taps detected in a 60 min period centered around individual epochs). Lastly, periods of tap inactivity which overlapped (by more than 25%) with the identified rest periods were classified as a sleep episode. Inactivity that lies outside of the potential rest periods were filtered out. Sleep periods exceeding 13 h were excluded to avoid misclassification of sleep from app disconnection which would have resulted in long periods of inactivity exceeding half a day. Sleep episodes recorded within the same day were concatenated to form a single sleep period if the total duration does not exceed 13 h. Otherwise, only the longest sleep episode was retained.

### Self-report through EMA

Subjects provided self-report data through an EMA app. EMA sessions were conducted twice daily, with each session taking a maximum of 5 min to complete. The window for completing Session 1 was from 08:00 AM till 12:00 PM (Session 1’s window was adjusted to 08:00 AM till 05:00 PM starting from 8th of June), and Session 2 from 08:00 PM till 12:00 AM. Subjects were prompted to provide their bed and wake times and subjective sleep quality (poor [1]–good [5]) from the previous night in Session 1 daily (and repeated in Session 2 if no response was recorded in Session 1). Self-reported sleep timings below 3 h or exceeding 13 h were removed as potential misreports and to ensure consistency across modalities. Furthermore, questions regarding users’ well-being (current mood: negative [0]–positive [100] and stress levels: not at all [0]–very stressed [100]) were included in both sessions. Short cognitive tasks^26^ were completed in Session 1 (data not presented here).

### Statistical analyses

From the wearable, phone, and self-report modalities, daily estimates of bedtime, wake time, midsleep time, and time in bed (TIB) were extracted. Furthermore, the Oura algorithm provides a daily estimate of WASO. These modalities were then compared for compliance rates, agreement, and discrepancies.

### Compliance

For each modality, the number of nights on which sleep data was recorded was counted. Overall compliance rates were calculated as well as their development over the 8-week monitoring period. To examine the effects of sleep modalities, week of participation, and weekday/weekend on compliance rate, a three-way repeated-measures analysis of variance (ANOVA) was conducted. Post-hoc analyses using Bonferroni correction were performed where significant interactions were observed.

### Agreement

To assess the agreement among sleep estimates obtained from the different modalities, pairwise Spearman’s rank correlation tests were conducted between the estimates from each modality with the mean of the other two modalities. Analyses were performed on the 7581 nights that had usable data from all three modalities. As the sleep estimates violated the normality assumption (see Supplementary Table 4), two-sided Wilcoxon signed-rank tests were then used to compare estimates obtained from Oura and tappigraphy against self-reported EMA to assess systematic discrepancies among modalities.

### Identification of high-discrepancy patterns based on sleep features

**Fig. 5:**
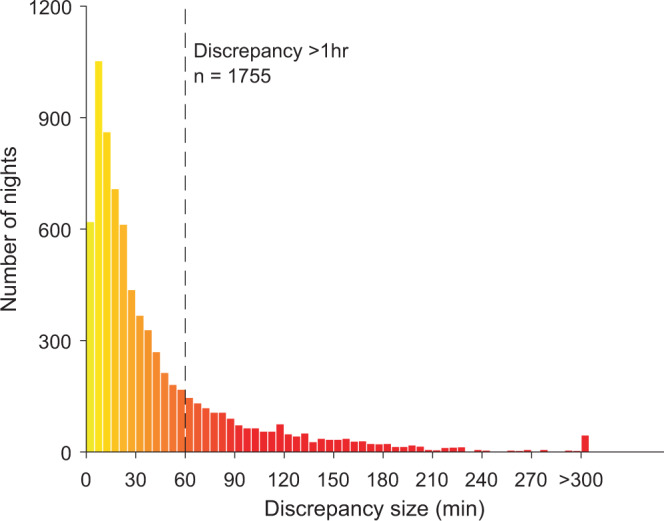
Histogram depicting the occurrence of discrepancies of a given magnitude (in minutes). For each night, the highest discrepancy among all three modalities was used. The dashed line indicates the discrepancy threshold of 1 h used in the analysis.

For nights with high discrepancy between modalities (>1 h; *n* = 1755; see Fig. 5), clustering analysis was performed with 13 sleep metrics used as features (4 features for each of the 3 modalities: [bedtime, wake time, midsleep time, and TIB], along with one feature only available from Oura [WASO]). To ensure that the features were weighted equally in the clustering process, all features were rescaled to vary from 0 to 1 using min–max normalization. Clustering was conducted using the *k*-means ++ algorithm on Matlab version R2017b (Mathworks, Natick, MA) for cluster center initialization paired with the squared Euclidean distance metric. To identify the optimal number of clusters appropriate for our dataset, we varied the number of clusters (*k* = 2–10) and assessed their associated within-cluster sums of squared distance. An optimal number of clusters was selected (*k* = 3) based on an assessment of the elbow plot while ensuring that the clusters obtained provided meaningful information for interpretation.

### Comparing individuals grouped by dominant discrepancy cluster

Individuals were grouped based on their dominant discrepancy pattern. Fourteen subjects did not have a clear dominant pattern (i.e., had an equal number of nights in their top two clusters) and were excluded from this analysis. Eight more subjects were excluded as they had no discrepancies larger than 1 h on any of the observed nights. To identify any characterizing features, the resulting groups were compared based on sleep metrics (on low-discrepancy nights), demographics, smartphone usage, and daily well-being, using one-way ANOVA and Pearson’s chi-squared test of independence. Kruskal–Wallis tests were used for cases where homogeneity of variance was violated. Statistical analyses were performed in Matlab version R2017b and R version 4.0.1 (R Core Team, 2020).

### Reporting summary

Further information on research design is available in the Nature Research Reporting Summary linked to this article.

## Supplementary information

Supplementary Information

Reporting Summary

## Data Availability

The data used in this study are available from the corresponding author upon reasonable request.

## Code availability

Statistical analyses were performed using standard packages in Matlab version R2017b and R version 4.0.1. Sleep estimates from the Tappigraphy modality were calculated using a previously published scoring algorithm^18^, and described in the “Methods” section.
