# Supplementary material for: Trait-like nocturnal sleep behavior identified by combining wearable, phone-use, and self-report data
Source: NPJ Digit Med. 2021 Jun 2;4:90. doi: 10.1038/s41746-021-00466-9 (PMC8172635; doi:10.1038/s41746-021-00466-9)
Supplement: Supplementary file 1 — Supplementary Information [file 41746_2021_466_MOESM1_ESM.pdf]

Supplementary Information

Trait-like Nocturnal Sleep Behavior Identified by Combining Wearable, Phone-use, and Self-report Data

Stijn A. A. Massar[1†], Xin Yu Chua[1†], Chun Siong Soon[1], Alyssa Ng[1], Julynn Ong[1], Nicholas Chee[1], Tih Shih Lee[2] Arko Ghosh[3], Michael W. L. Chee[1]*

[1] Sleep and Cognition Laboratory, Centre for Sleep and Cognition, Yong Loo Lin School of Medicine, National University of Singapore

[2] Laboratory of Neurobehavioral Genomics, Neuroscience and Behavioral Disorders Programme, Duke-NUS Medical School, Singapore

[3] Institute of Psychology, Leiden University, Leiden, the Netherlands

† These authors contributed equally

* Corresponding author

Michael W.L. Chee, Centre for Sleep and Cognition, Yong Loo Lin School of Medicine, 12 Science Drive 2, National University of Singapore, Singapore 117549.
Email: michael.chee@nus.edu.sg.

Assessing stability of obtained clusters across discrepancy thresholds

To assess the stability of the k = 3 cluster solution found for high-discrepancy nights (Supplementary Figure 1a & b), we repeated the clustering of nights at different discrepancy thresholds (1.5, 2, and 2.5 hr). Resulting clusters were very similar to the original solution, both in cluster configuration, and in the optimal number of clusters (Supplementary Figure 1c & d). Furthermore, grouping of individuals based on their dominant cluster pattern remained stable across thresholds, with all overlap coefficients[1] > .74 (Supplementary Table 1).

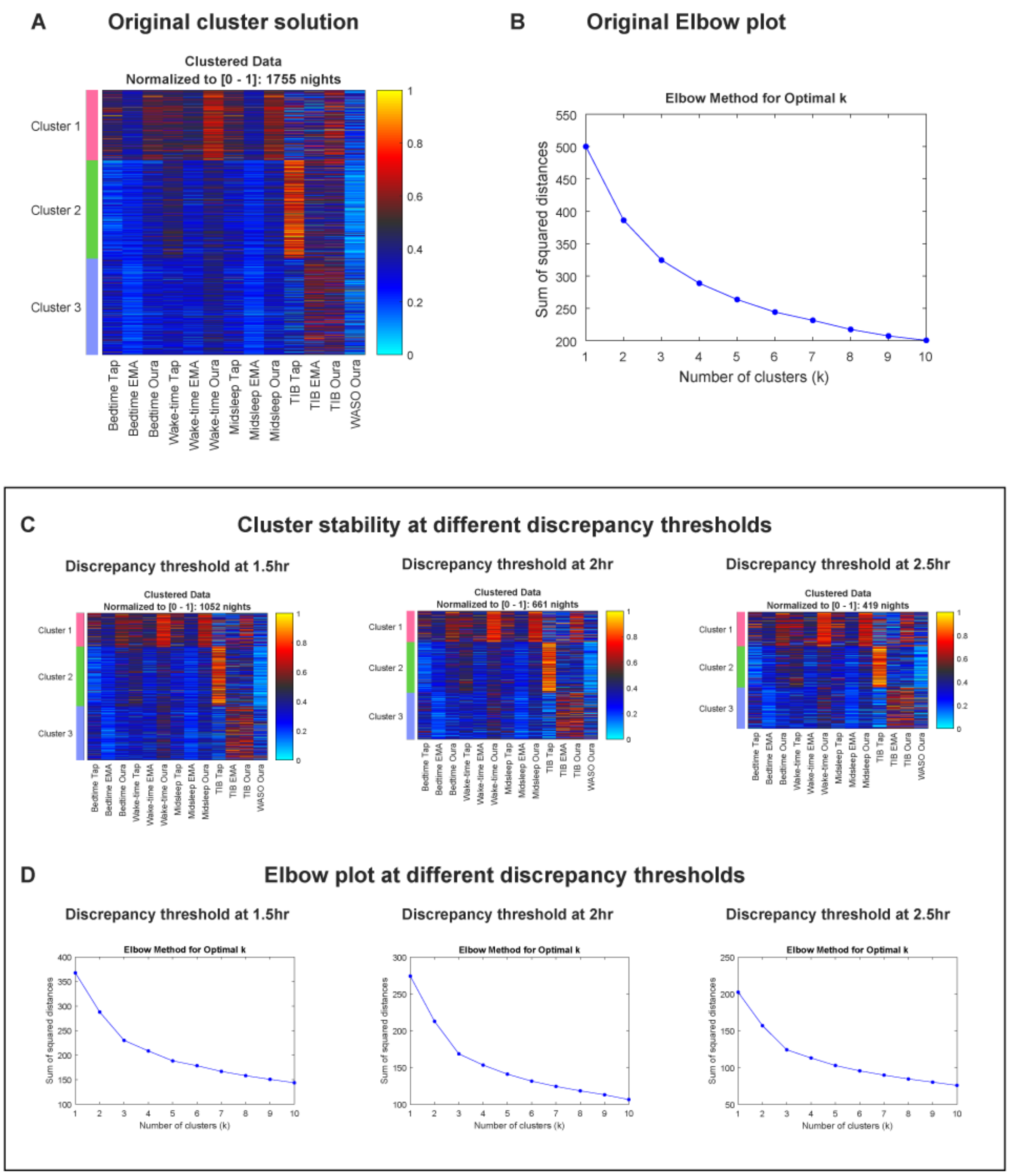

Supplementary Figure 1. Clustering solutions and elbow plots indicating optimal cluster number (a&b) for the original cluster (discrepancy threshold = 1hr), and (c&d) controls at different discrepancy thresholds 1.5, 2, and 2.5hr (bar heights differ as fewer nights are included at higher thresholds).

Supplementary Table 1. Stability of discrepancy-cluster groups for clustering at different thresholds

	Discrepancy threshold		
	1.5hr	2hr	2.5hr
Overlap coefficient	.84	.79	.74
Subjects (N)†	142	122	100

† Number of subjects varies across thresholds as fewer discrepant nights are included in clustering at higher discrepancy thresholds.

The distribution of cluster types of high-discrepancy nights showed that for most individuals a clear dominant pattern was found (Supplementary Figure 2). Mean and median percentages of nights in the dominant cluster were above 76% for all groups (Supplementary Table 2).

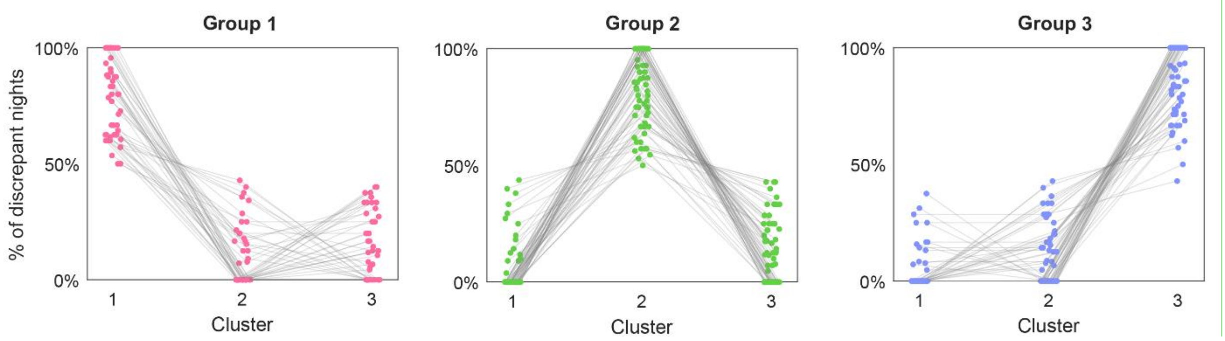

Supplementary Figure 2. Percentages of high-discrepancy nights for each cluster type displayed for each individual.

Supplementary Table 2. Percentages of high-discrepancy nights (mean, median, and interquartile range) for each cluster type per group.

	Cluster 1			Cluster 2			Cluster 3		
	Mean	Median	IQR	Mean	Median	IQR	Mean	Median	IQR
Group 1	**76.6%**	**78.6%**	**26.7%**	9.5%	0%	17.6%	13.9%	11.8%	26.7%
Group 2	5.2%	0%	0%	**82.7%**	**85.2%**	**31.8%**	12.1%	7.4%	22.2%
Group 3	4%	0%	0%	9.6%	0%	16.7%	**86.4%**	**91.3%**	**26.0%**

Bold faced numbers indicate the dominant cluster type for each group.

Supplementary Table 3. Overview of practical characteristics of the three methods used

	**Wearable (Oura ring)**	**Phone (Tappigraphy)**	**Self-report (EMA)**
**Availability**	• Consumer device	• Android phones only	• Android phones only†
**Scalability**	• Moderate<br>• Depends on physical device availability	• High<br>• Uses participant's own phone	• High<br>• Uses participant's own phone
**Participant effort**	• Participant needs to wear the device to sleep<br>• Regular syncing of data with app<br>• Regular charging of device	• No active participant input needed<br>• Background app<br>• Passive phone use monitoring	• Needs daily input from participant
**Sleep metrics**	• Bedtime, Wake time, Time-in-Bed<br>• Sleep staging<br>• Wake-after sleep-onset, Sleep efficiency	• Bedtime, Wake time, Time-in-Bed	• Bedtime, Wake time, Time-in-Bed<br>• Subjective sleep quality
**Other metrics**	• Physical activity<br>• Heart rate<br>• Heart rate variability	• Phone use time<br>• Screen interactions<br>• App use	• Wellbeing questions<br>• Cognitive assessment

† EMA application was developed in-house. Restriction to Android phones reflects the availability at the time of study. An iOS version is currently being developed in-house. Several off-the-shelf alternatives are available commercially or open source.

Supplementary Table 4. Kolmogorov-Smirnov test of normality with Lilliefors Correction

		Statistic	p
Bedtime	Oura	.044	<.001
	Tap	.039	<.001
	EMA	.089	<.001
	Mean (Tap & EMA)	.043	<.001
	Mean (Oura & EMA)	.049	<.001
	Mean (Tap & Oura)	.035	<.001
Wake time	Oura	.057	<.001
	Tap	.045	<.001
	EMA	.074	<.001
	Mean (Tap & EMA)	.044	<.001
	Mean (Oura & EMA)	.051	<.001
	Mean (Tap & Oura)	.052	<.001

n = 7581

References

1 Szymkiewicz, D. Une conlribution statistique à la géographie floristique. *Acta Societatis Botanicorum Poloniae* **11**, 249-265 (1934).
