# Supplementary material for: Trait-like nocturnal sleep behavior identified by combining wearable, phone-use, and self-report data
Source: NPJ Digit Med. 2021 Jun 2;4:90. doi: 10.1038/s41746-021-00466-9 (PMC8172635; doi:10.1038/s41746-021-00466-9)
Supplement: Supplementary file 2 — Reporting Summary [file 41746_2021_466_MOESM2_ESM.pdf]

nature research

Corresponding author(s): Michael Chee

Last updated by author(s): Mar 31, 2021

Reporting Summary

Nature Research wishes to improve the reproducibility of the work that we publish. This form provides structure for consistency and transparency in reporting. For further information on Nature Research policies, see our Editorial Policies and the Editorial Policy Checklist.

Statistics

For all statistical analyses, confirm that the following items are present in the figure legend, table legend, main text, or Methods section.

n/a	Confirmed	
☐	☒	The exact sample size (*n*) for each experimental group/condition, given as a discrete number and unit of measurement
☐	☒	A statement on whether measurements were taken from distinct samples or whether the same sample was measured repeatedly
☐	☒	The statistical test(s) used AND whether they are one- or two-sided<br>*Only common tests should be described solely by name; describe more complex techniques in the Methods section.*
☒	☐	A description of all covariates tested
☐	☒	A description of any assumptions or corrections, such as tests of normality and adjustment for multiple comparisons
☐	☒	A full description of the statistical parameters including central tendency (e.g. means) or other basic estimates (e.g. regression coefficient) AND variation (e.g. standard deviation) or associated estimates of uncertainty (e.g. confidence intervals)
☐	☒	For null hypothesis testing, the test statistic (e.g. *F*, *t*, *r*) with confidence intervals, effect sizes, degrees of freedom and *P* value noted<br>*Give P values as exact values whenever suitable.*
☒	☐	For Bayesian analysis, information on the choice of priors and Markov chain Monte Carlo settings
☒	☐	For hierarchical and complex designs, identification of the appropriate level for tests and full reporting of outcomes
☒	☐	Estimates of effect sizes (e.g. Cohen's *d*, Pearson's *r*), indicating how they were calculated

Our web collection on statistics for biologists contains articles on many of the points above.

Software and code

Policy information about availability of computer code

Data collection	Sleep and activity information were collected through a wearable (Oura Ring Heritage; Oura Health Oy, Oulu, Finland). Sleep estimates were processed by Oura Health's proprietary algorithm.<br>Smartphone touch screen interactions were collected through the TapCounter mobile app (version 1.2.6), Quantactions, Lausanne, Switzerland (https://quantactions.com/)<br>Self-report data were collected through a custom made Ecological Momentary Assessment (EMA) application, Z4IP mobile app (version 1.2.2)
Data analysis	Statistical analyses were performed using standard packages in Matlab version R2017b and R version 4.0.1.<br>The algorithm used for sleep detection through touchscreen interactions (tappigraphy) has previously been published by Borger & Ghosh, 2019.<br>Borger, J. N., Huber, R. & Ghosh, A. Capturing sleep-wake cycles by using day-to-day smartphone touchscreen interactions. NPJ Digit Med 2, 73, doi:10.1038/s41746-019-0147-4 (2019).

For manuscripts utilizing custom algorithms or software that are central to the research but not yet described in published literature, software must be made available to editors and reviewers. We strongly encourage code deposition in a community repository (e.g. GitHub). See the Nature Research guidelines for submitting code & software for further information.

Data

Policy information about availability of data

All manuscripts must include a data availability statement. This statement should provide the following information, where applicable:

- Accession codes, unique identifiers, or web links for publicly available datasets
- A list of figures that have associated raw data
- A description of any restrictions on data availability

The data used in this study are available from the corresponding author upon reasonable request.

Field-specific reporting

Please select the one below that is the best fit for your research. If you are not sure, read the appropriate sections before making your selection.

☐ Life sciences ☒ Behavioural & social sciences ☐ Ecological, evolutionary & environmental sciences

For a reference copy of the document with all sections, see nature.com/documents/nr-reporting-summary-flat.pdf

Behavioural & social sciences study design

All studies must disclose on these points even when the disclosure is negative.

Study description	Observational study quantitative data.
Research sample	Two hundred staff and students from the National University of Singapore were recruited for the study. After two subjects withdrew from the study, we had a remaining sample of 198 (mean age = 26.20 years, 61 males, 78 staff).
Sampling strategy	The study includes a convenience sample of staff and students from the National University of Singapore. No a priori power analysis was performed. Sample size target was set as the largest possible sample given device (Oura ring) availability (N=200).
Data collection	Sleep data was collected through three separate modalities:<br>(1) a sleep and activity tracking ring, Oura ring<br>(2) a smartphone app tracking touchscreen interactions<br>(3) an ecological momentary app for daily self-reports
Timing	27 April till 12 July 2020.
Data exclusions	Out of 200 initial participants, two subjects withdrew. All resulting N=198 participants were included in the primary analyses (i.e. compliance, modality agreement, discrepancy). Twenty-two participants were excluded from the discrepancy-group analysis (N=14 had no clear predominant pattern, N=8 had no nights with discrepancies >1h)
Non-participation	Two subjects withdrew mid-study.
Randomization	Not applicable.

Reporting for specific materials, systems and methods

We require information from authors about some types of materials, experimental systems and methods used in many studies. Here, indicate whether each material, system or method listed is relevant to your study. If you are not sure if a list item applies to your research, read the appropriate section before selecting a response.

Materials & experimental systems

n/a	Involved in the study	
☒	☐	Antibodies
☒	☐	Eukaryotic cell lines
☒	☐	Palaeontology and archaeology
☒	☐	Animals and other organisms
☐	☒	Human research participants
☒	☐	Clinical data
☒	☐	Dual use research of concern

Methods

n/a	Involved in the study	
☒	☐	ChIP-seq
☒	☐	Flow cytometry
☒	☐	MRI-based neuroimaging

Human research participants

Policy information about studies involving human research participants

Population characteristics	See above
Recruitment	Participants were recruited through word-of-mouth and research advertisements posted on our university research recruitment platform for both students and staff.
Ethics oversight	All procedures were approved by the Institutional Review Board of the National University of Singapore (NUS-IRB Ref Code: N-20-039), and all participants signed written informed consent before commencing the study.

Note that full information on the approval of the study protocol must also be provided in the manuscript.
